# Genetic characterization of foot-and-mouth disease virus in cattle in Northern Egypt

**DOI:** 10.14202/vetworld.2025.238-248

**Published:** 2025-01-30

**Authors:** Sarah G. Yousef, Hend M. El Damaty, Hussein A. Elsheikh, Yousry A. El-Shazly, Eman Metwally, Samar Atwa

**Affiliations:** 1 Infectious Diseases, Department of Animal Medicine, Faculty of Veterinary Medicine, Zagazig University, 44511, Zagazig, Sharkia, Egypt; 2 Veterinary Hospital, Faculty of Veterinary Medicine, Zagazig University, 44511, Zagazig, Sharkia, Egypt; 3 Department of Internal Medicine and Infectious Diseases, Faculty of Veterinary Medicine, Mansoura University, 35516, Mansoura, Dakahlia, Egypt; 4 Department of Veterinary Clinical Sciences, Faculty of Veterinary Medicine, Jordan University of Science and Technology, Irbid 22110, Jordan

**Keywords:** foot-and-mouth disease, Egypt, cattle, FMDV serotypes, *VP1* sequencing, vaccine efficacy

## Abstract

**Background and Aim:**

Foot-and-mouth disease (FMD) is a highly contagious viral disease that poses significant economic threats to livestock globally. This study aimed to confirm the presence of FMD virus (FMDV) in Egyptian cattle and identify the predominant serotypes contributing to outbreaks in Sharkia and Dakahlia provinces in 2022.

**Materials and Methods:**

A total of 65 cattle showing acute FMD symptoms were sampled. Desquamated epithelial tissues and oral secretions were analyzed using reverse transcriptase polymerase chain reaction with universal and serotype-specific primers. Seven representative samples underwent sequencing for phylogenetic and genetic variability analysis.

**Results:**

All sampled animals tested positive for FMDV. Serotype A accounted for 72.3% of cases, while 27.7% were serotype O. Sequence analysis identified FMDV serotype A (African topotype, genotype IV) and serotype O (East Africa-3 topotype) as the outbreak-causing strains. The identified strains exhibited significant genetic divergence from the vaccine strains used in Egypt, with notable amino acid substitutions in the VP1 protein’s G-H loop. These mutations raise concerns about the efficacy of existing vaccines against current field strains.

**Conclusion:**

The study highlights the ongoing threat of FMD in Egypt, particularly among smallholder cattle farmers. The genetic divergence between circulating FMDV and vaccine strains underscores the need to continuously monitor and update vaccine formulations to enhance disease control efforts. Implementing stricter animal movement regulations and tailored vaccination strategies is essential for effective management.

## INTRODUCTION

Foot-and-mouth disease (FMD) is one of the most infectious and economically significant viral diseases affecting animal health and production. The far-reaching impact of FMD on the universal trade of live cloven-hoofed animals and their by-products cannot be overstated, making it imperative to address the FMD burden with urgency and vigilance [1]. The FMD virus (FMDV) is a causative agent classified into seven serotypes [2]. These serotypes, while causing similar clinical manifestations, are antigenically distinct, and immunity to one serotype does not confer protection against others [3]. The *VP1* gene on the virus’s surface exhibits significant variability and includes serotype-specific amino acid sequences, and it is used for serotype identification [4]. The primary agricultural animals affected by the disease are cattle, swine, sheep, and goats. Cattle are especially crucial due to their high susceptibility and role as the primary source of FMDV transmission [5].

During the last few years, several outbreaks caused by FMDV serotypes A, O, and SAT-2 have occurred among livestock in Egypt, resulting in significant losses among cattle [6]. The first official report of FMD in Egypt dates back to 1950, involving serotypes O and SAT-2 [7, 8]. Serotype SAT-2 disappeared after 1950 and was later isolated in an outbreak in 2012 and included two novel strains closely linked to the 2008 Sudan strain [9, 10]. FMDV serotype A was reported in 1952, disappeared in 1976, and re-emerged in 2006 due to the importation of live animals from Ethiopia [11]. Reports by El Nahas and Salem [12] and El Damaty *et al*. [13] have also identified new lineages from serotypes O and SAT2 topotypes, which are unusually associated with higher mortality rates in young and adult animals. This is noteworthy; FMD typically does not cause significant fatalities in adult animals, but it causes substantial economic losses, significantly impacting the livelihoods and incomes of affected farmers in smallholder production systems [14]. Despite compulsory vaccination in Egypt, the country continues to face challenges related to FMD outbreaks [13, 15]. Livestock that is usually infected displays clinical signs such as high body temperature, cessation of rumination, increased salivation, formation of sores on the lips, tongue, mouth, nose, between the toes, and sometimes on the skin of the teats, and reduced milk yield [5].

In this study, we focused on the smallholder production system, a prevalent method of livestock production in Egypt. Unlike most FMD studies, which focus on farms with intensive production systems [15–17], our research sheds light on small herds’ vulnerability to subsistence household incomes [18, 19]. Due to budgetary constraints, the absence of proper hygiene and biosecurity measures puts production systems at high risk of FMD outbreaks, leading to rapid disease spread and severe consequences for smallholders. This underscores the urgent need for attention and support to this vulnerable sector. Hence, this study aimed to confirm the existence of FMDV and identify the most predominant serotypes associated with outbreaks among small-scale cattle breeders in two of Egypt’s major agricultural provinces in 2022.

## MATERIALS AND METHODS

### Ethical approval

The study protocol was officially approved by the Institutional Animal Care and Use Committee (IACUC) of Zagazig University, Egypt (approval number ZU-IACUC/2/F//455/2022).

### Study period and location

The study was conducted from April to August 2022 in the Sharkia and Dakahlia in the Eastern Nile Delta of northern Egypt, which experienced FMD outbreaks with considerable losses among cattle. These provinces are among the most important provinces in Egypt for livestock production.

### Study population

Sixty-five cattle aged 4–7 years, including 39 females and 26 males, were randomly chosen for this study. These animals were from different villages and were part of a semi-intensive farming program. Cattle were clinically examined according to Constable *et al*. [20] method. Cattle were selected based on reported symptoms of FMD involving vesicle formation in the mouth and foot, high fever, and inappetence. In Egypt, animal vaccination depends on locally produced vaccines, specifically the inactivated polyvalent official vaccines manufactured by the Veterinary Serum and Vaccine Research Institute (Cairo, Egypt), which include (O/EGY/15BH-2009, A/IRN/1/2005, A/EGY1/2012, SAT-2 EGY/H1Ghb/2012, and SAT-2 Lib-12) strains of FMDV serotypes O, A, and SAT-2, respectively.

### Samples collection

Sixty-five desquamated epithelial and saliva samples were collected from the mouths of clinically suspected FMD cases in cattle. These samples were placed in a transport medium (BioWhittaker, Lonza Group Ltd., Switzerland) comprising equal volumes of glycerol and phosphate-buffered saline (pH 7.2–7.6) with 2% antibiotic-antimycotic. The samples were promptly transported to the laboratory on ice (4°C) [21].

### RNA extraction and serotyping of FMDV

RNA was extracted from the collected samples according to the manufacturer’s recommendations using the QIAamp MiniElute Virus Spin Kits (Qiagen, GmbH, Germany). In brief, 0.2 mL of the sample suspension was incubated at 56°C for 15 min with 0.025 mL of Qiagen protease and 0.2 mL of AL lysis buffer; then 0.25 mL of pure ethanol was added to the lysate. The sample was washed and centrifuged, and the nucleic acid was eluted with 0.1 mL of the elution buffer. The oligonucleotide primer pairs (Metabion, Germany, GmbH) used in the reverse transcriptase polymerase chain reaction (RT-PCR) assays are listed in Table 1 [10, 22, 23]. In a PTC-100 TM programmable thermal cycler (MJ Research Inc., Waltham, USA), PCR amplification was conducted with a final volume of 0.025 mL of the subsequent reaction mixture: 12.5 μL of Quantitect probe buffer RT-PCR (Qiagen, GmbH), 1 μL of each primer (20 pmol), 4.25 μL of water, 0.25 μL of reverse transcriptase-enzyme, and 6 μL of template DNA. Reverse transcription was applied at 50°C for 30 min; an initial denaturation was done at 95°C for 5 min, followed by 35 cycles of 94°C for 30 s., 55°C for 30 s (for FMD A and O), and 60°C for 40 s for SAT-2 and 72°C for 30 s. The final extension was performed at 72°C for 12 min. Subsequently, the PCR products were subjected to electrophoresis on a 1% agarose gel (AppliChem, Germany, GmbH) alongside negative and positive controls representing various reference FMD serotypes (reference serotype O strain; O/EGY/3/93 [EU553840.1]; reference serotype A strain; A/EGY/1/2006 [EF208757.1]; and reference serotype SAT-2 strain; SAT-2/EGY/9/2012 [JX014255.1]). The controls were supplied by the Biotechnology Unit at the Animal Health Research Institute in Dokki, Giza, Egypt.

**Table 1:** Primer sequences, target genes, and amplicon sizes used to characterize FMDV.

Serotype	Target gene	Primer sequence (5’– 3’)	Product size (bp)	Reference
FMD	5’Untranslated Region (UTR)	F: GCCTGGTCTTTCCAG GTCT.	326	[22]
		R: CCAGTCCCCTTCTCAGATC		
FMD A	VP3-2B	F: TACCAAATTACACACGGGAA	863-866	[10]
		R: GACATGTCCTCCTGCATCTG		
FMD O	VP3-2B	F: ACCAACCTCCTTGATGTGGCT	1301	
		R: GACATGTCCTCCTGCATCTG		
FMD SAT-2	VP3-2B	F: GAACTACCACTTCATGTACACAG	1279	[23]
		R: ACAGCGGCCATGCACGACAG		

FMD=Foot-and-mouth disease, FMDV=Foot-and-mouth disease virus, FMDV A=Foot-and-mouth disease virus serotype A, FMDV O=Foot-and-mouth disease virus serotype O

### DNA sequencing and sequence analysis

Seven PCR products, representing samples from diseased cattle, were chosen for DNA sequencing using an Applied Biosystems 3130 genetic analyzer (Hitachi, Tokyo, Japan). These samples were selected based on two criteria: the distinct clinical signs exhibited by the cattle and the well-defined gel bands observed during the initial PCR analysis. The amplified DNA fragments were purified with the QIAquick PCR purification kit (Qiagen, Courtaboeuf, France). A BigDye Terminator V3.1 cycle sequencing kit (PerkinElmer Inc., Waltham, MA, USA) was used for the sequencing reaction and purification using Centri-Sep spin columns. The sequences were initially submitted to BLAST^®^ analysis (Basic Local Alignment Search Tool) to assess the sequence identity to GenBank accessions from the National Center for Biotechnology Information (NCBI) database (NCBI, www.ncbi.nlm.nih.gov/BLAST/) [24]. The distance matrix and neighbor-joining phylogenetic tree were constructed using MEGA11 software with a bootstrap trial 1000 [25]. The nucleotide sequences were translated using the ExPASy (Expert Protein Analysis System) Translate Tool (http://us.expasy.org/, Swiss Institute of Bioinformatics SIB, Geneva, Switzerland). The amino acid sequences were aligned using BioEdit software (version 7.1) [26]. The nucleotide sequences of the partial *VP1* gene generated in this study were deposited in GenBank under accession numbers OP321260.1-OP321265.1 regarding serotype A and OP293055.1 for serotype O (Table 2). The metadata collected for reference sequences of the *VP1* gene of FMDV serotypes A and O were restored from GenBank and are listed in Tables 3 and 4.

**Table 2:** Serotypes and GenBank genomic sequence accession numbers of the FMDV strains identified in this study.

Animal	Sequence name	GenBank accession numbers
Cattle	FMDV A/Sharkia 1/EGY/2022	OP321260.1
Cattle	FMDV A/Sharkia 2/EGY/2022	OP321261.1
Cattle	FMDV A/Dakahleya 3/EGY/2022	OP321262.1
Cattle	FMDV A/Sharkia 4/EGY/2022	OP321263.1
Cattle	FMDV A/Sharkia 5/EGY/2022	OP321264.1
Cattle	FMDV A/Sharkia 6/EGY/2022	OP321265.1
Cattle	FMDV O/Sharkia 7/EGY/2022	OP293055.1

FMDV A: Foot-and-mouth disease virus serotype A, FMDV O: Foot-and-mouth disease virus serotype O

**Table 3:** Details of the isolated FMDV serotype A and those retrieved from GenBank whose sequence was analyzed and compared in the current study.

FMDV strain	Topotype	Origin	GenBank Accession number
A/Sharkia 1/EGY/2022	African/G-IV	Egypt	−OP321260.1
A/Sharkia 2/EGY/2022	African/G-IV	Egypt	−OP321261.1
A/Sharkia 3/EGY/2022	African/G-IV	Egypt	−OP321262.1
A/Sharkia 4/EGY/2022	African/G-IV	Egypt	−OP321263.1
A/Sharkia 5/EGY/2022	African/G-IV	Egypt	−OP321264.1
A/Sharkia 6/EGY/2022	African/G-IV	Egypt	−OP321265.1
A/EGY 1/2012	Asian	Egypt	KC440882.1
A/Egypt/2006	African/G-VII	Egypt	JF749843.1
A/Egy/Damietta/2015	Asian	Egypt	MG552838.1
A/IRN/1/2005	Asian	Egypt	EF208769.1
A/EGY/1/2006	African/G-V II	Egypt	EF208757.1
A/Egy/Dakahlia/2020	African/G-IV	Egypt	OL456140.1
A/Egy/AHRI-RV39/2020	African/G-IV	Egypt	MW413347.1
A/Egy/AHRI-RL382-Ven/2022	Euro-SA	Egypt	OP131710.1
A/SUD/3/77	African/G-IV	Sudan	GU566064.1
A/ETH/19/2015	African/G-IV	Ethiopia	MN987497.1
A/Ismailia 2/Egy/2016	African/G-IV	Egypt	KX446997.1
A/Damietta/Egypt/2016	African/G-IV	Egypt	MT863266.1
A/SUD/6/2018	African/G-IV	Sudan	MK422591.1
A/EGY/1/72	African/G-II	Egypt	KF112901.1
A/a21kenya iso77	African/G-III	Kenya	AY593761.1
A/UGA/13/66	African/G-VI	Uganda	KF561705.1
A/KEN/K91/2015	African/G-I	Kenya	MH882568.1
A/KEN/K74/2016	African/G-I	Kenya	MH882567.1
A/IRN/10/2003	Asian	Iran	KF112905.1
A/IRN/15/2007	Asian	Iran	FJ755061.1
A/Kafr El-Sheikh6/Egypt/2022	African/G-IV	Egypt	OQ316633.1
A/EGY/Behiera/2022	African/G-IV	Egypt	OR989997.1
A/EGY/Kafr El-Sheikh/2022	African/G-IV	Egypt	OR989996.1

**Table 4:** Details of the isolated FMDV serotype O and those retrieved from GenBank whose sequence was analyzed and compared in the current study.

FMDV strains	Topotype	Origin	GenBank Accession number
O/Sharkia7/Egypt/2022	EA-3	Egypt	−OP293055.1
O Lahore vaccine	ME-SA	Egypt	EU244455.1
O/EGY/15BH-2009	ME-SA	Egypt	JQ837836.1
O/HKN/16/96	Cathay	Hong Kong	AJ294923.1
O/KEN/83/79	EA-1	Kenya	AJ303511.1
O/Egy/Faiyum/2015	EA-3	Egypt	MG552845.1
O/Egy/Dakahlia/2017	EA-3	Egypt	MG552848.1
O/Ismailia 5/Egy/2016	EA-3	Egypt	KX447128.1
O/Giza1/Egy/2017	EA-3	Egypt	MF322682.1
O/ISA/9/74	ISA-1	Indonesia	AJ303502.1
O/JAV/5/72	ISA-2	Indonesia	AJ303509.1
O/3/Venezuela/51	Euro-SA	Venezuela	AJ004645.1
O/SUD/2/2017	EA-3	Sudan	MK422564.1
O/Egypt 3SD	EA-3	Egypt	ON455107.1
O/Egypt 5SD	EA-3	Egypt	ON455108.1
O/Dakahlia/Egypt/2014	ME-SA	Egypt	KP940473.1
O/ISR/17/2007	ME-SA	Israel	MT443435.1
O/OUGA2008KAMULI	EA-2	Uganda	JN974312.1
O/ETH/58/2005	EA-4	Ethiopia	MH053312.1
O/ETH/5/2019	EA-3	Ethiopia	MN987484.1
O/Giza 2/Egy/2016	EA-3	Egypt	KX447132.1
O/EGY/13/2015	EA-3	Egypt	OM221200.1
O/Egypt/35/2016	EA-3	Egypt	MG925050.1
O/Giza 1/Egy/2016	EA-3	Egypt	KX447131.1
O/SUD/1/2016	EA-3	Sudan	MK422563.1
O/EGY/19/2012	EA-3	Egypt	MK422548.1
O/Dakahlia/Egypt/2014	ME-SA	Egypt	KP940473.1
O/TUR/14/2007	ME-SA	Turkey	MT443803.1
O/CIV/8/99	WA	Cote d’Ivoire	AJ303485.1
O/ETH/58/2005	EA-4	Ethiopia	KJ831671.1
O/ISA/8/83	ISA-1	Indonesia	AJ303503.1
O/ISA/1/74	ISA-2	Indonesia	AJ303501.1
O/o1campos94 iso94	Euro-SA	Argentina	AY593819.1

East Africa 1 (EA-1), East Africa 2 (EA-2), East Africa-3 (EA-3), Middle-East South Asian (ME-SA), Europe-South America (EURO-SA), Cathay, Indonesia-1 (ISA-1), Indonesia-2 (ISA-2), and West Africa (WA) topotypes.

## RESULTS

### Clinical signs of FMDV infection in animals

**Figure 1:**
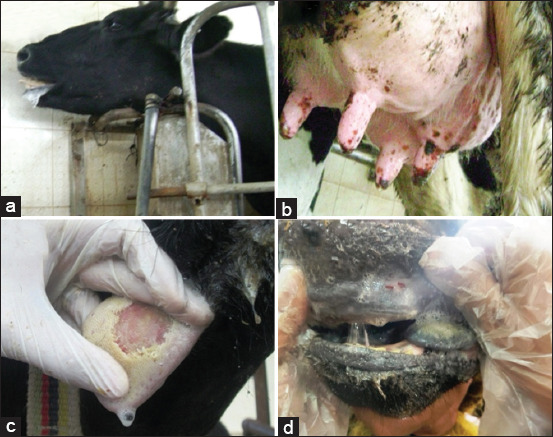
Cattle presenting clinical signs of FMD in Egypt. (a) Salivation, (b) Blisters on teats, (c) Erosion on the tongue, and (d) Ulcer formation of the dental pad. FMD=Foot-and-mouth disease.

The FMDV-infected cattle exhibited typical FMD symptoms, including fever ranging from 39°C to 40°C and reduced appetite. They also experienced excessive salivation and had blister-like sores in their mouths, tongues, and the skin of the udder and teat openings (Figures 1a and b). Furthermore, there was a hemorrhage at the corners of the mouth, likely resulting from or associated with existing ulcers in the tongue and the dental pad (Figures 1c and d). Some of the blisters burst, leaving painful erosions. Some calves died suddenly without any prior signs of illness.

### FMDV detection and serotyping

The results confirmed that all 65 sampled cattle displaying clinical signs suggestive of FMD were positive for the different serotypes tested using real-time RT-PCR. Of the isolates, 47 (72.3%) were serotype A and 18 (27.7%) were serotype O. No SAT-2 serotype was detected. In addition, *VP1* coding sequences were obtained for the seven virus isolates six, of which were serotypes A and O.

### *VP1* phylogenetic and amino acid sequence analyses of FMDV serotype A

**Figure 2:**
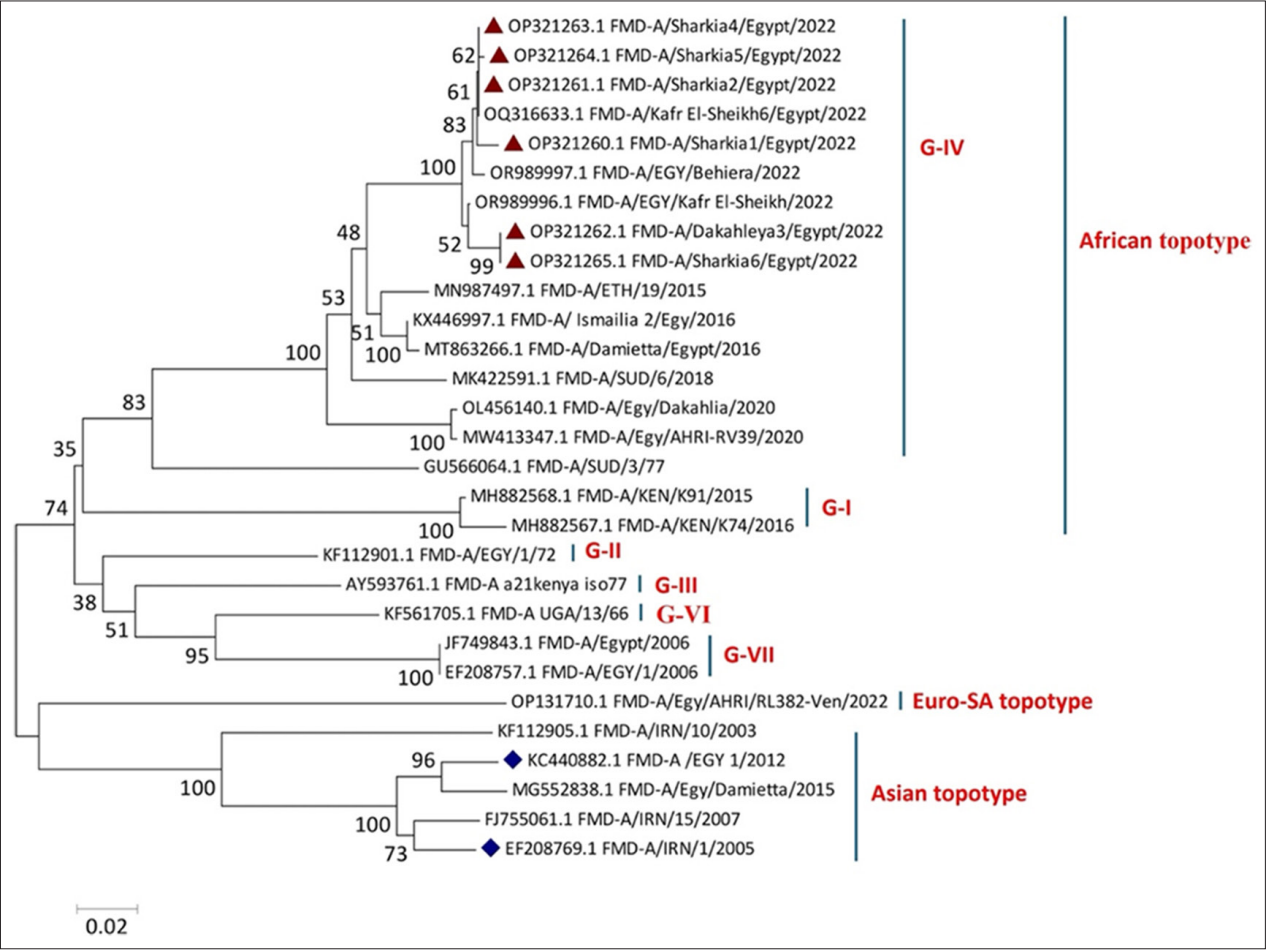
Phylogenetic analysis of the *VP1*-coding sequences shows the relationships among different FMDV serotype A of various topotypes. The tree was constructed using MEGA11 software using the Tamura-Nei model of nucleotide substitution with 1000 bootstrap replicates. The colored triangles represent the FMD strains identified in this study. FMD=Foot-and-mouth disease.

**Figure 3:**
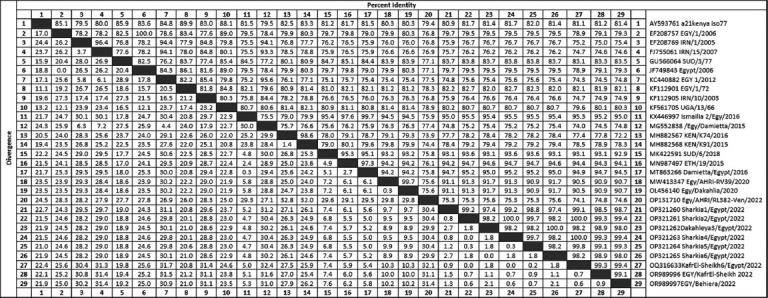
Nucleotide sequence distance matrix shows the percentage identity and divergence among the FMDV serotype A strains identified in this study and those retrieved from the National Center for Biotechnology Information database (https://www.ncbi.nlm.nih.gov/pubmed/). FMDV=Foot-and-mouth disease virus.

In this study, *VP1*-coding sequencing and phylogenetic analysis demonstrated significant relatedness among the outbreak-causing strains (n = 6), ranging from 98% to 99.4% nucleotide identity, which shared a common ancestor with the reference prototype African G IV (FMD-A/SUD/3/77; GU566064.1). In addition, they were grouped separately from the other two topotypes identified in Egypt. Our genomic sequences from the outbreak strains of the year 2022 (OP321260.1-OP321265.1) showed close relatedness to the African strains (A/ETH/19/2015; MN987497.1) and (A/SUD/6/2018; MK422591.1) reported in the 2015 and 2018 outbreaks in Ethiopia and Sudan, respectively (Figure 2). This confirmed that serotype A (African topotype and genotype IV [Africa/G-IV]) was responsible for the 2022 FMD outbreak in the two major governorates of northern Egypt. Furthermore, the sequence distance matrix (Figure 3) revealed high similarity, ranging from 98% to 100%, between the sequences generated in this study and those of other strains that circulated in Egypt during the 2022 outbreaks (A/KafrEl-Sheikh6/Egypt/2022[OQ316633.1], A/EGY/Behiera/2022 [OR989997.1], and A/EGY/KafrEl-Sheikh/2022[OR989996.1]). The current strains were closely related to the Egyptian strains isolated between 2015 and 2016 (A/ETH/19/2015 [MN987497.1]; A/Ismailia 2/Egy/2016[KX446997.1] and A/Damietta/Egypt/2016 [MT863266.1]), with an average similarity of 95%, except for the strain FMD-A/Egy/Damietta/2015 Accession No. (MG552838.1), which clustered with the Asian topotype with a 24.6%–25.2% variance. Additionally, our strains showed 8.3%–9.1% variance with Egyptian strains of 2020 (A/Egy/AHRI-RV39/2020[MW413347.1] and A/Egy/Dakahlia/2020 [OL456140.1]).

**Figure 4:**
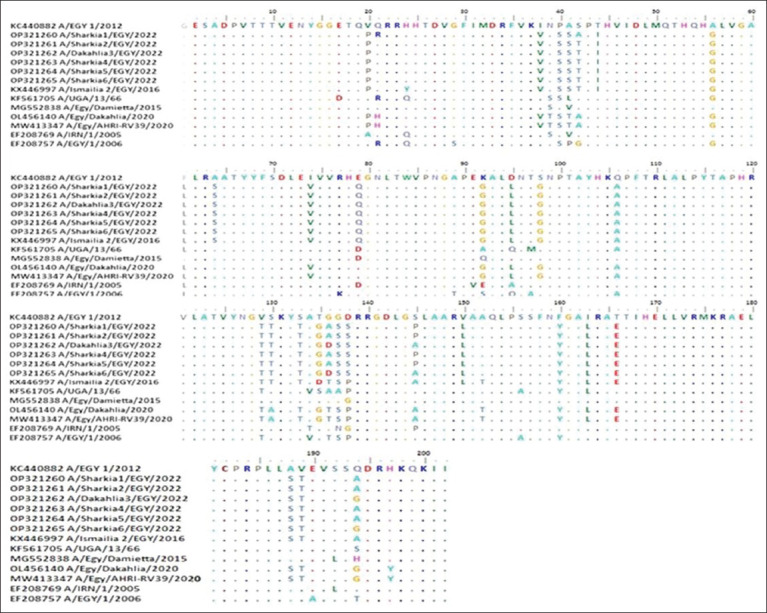
Alignment and comparison of the first 200 deduced amino acids of the *VP1* gene of the FMD serotype A strains identified in this study and those of the vaccine strain. The identity of the vaccine strain is indicated by dot-filled lines. FMD=Foot-and-mouth disease.

Multiple sequence alignment of Egyptian FMDV serotype A from different outbreaks revealed differences in amino acid sequences, especially in the G-H loop (135-160), for each outbreak strain. Comparing the amino acid sequences of the G-H loop with the vaccine strain (A/EGY 1/2012 [KC440882.1]), the study strains had seven amino acid substitutions in (G-H Loop) compared with the vaccine strain, with a 28% difference. These amino acid substitutions were performed in three situations: 135TGGD138, which was changed to 135GASS138 or 135GDSS138, S146P/A, and V151L (Figure 4).

### *VP1* phylogenetic analysis and amino acid sequences of the FMDV serotype O

**Figure 5:**
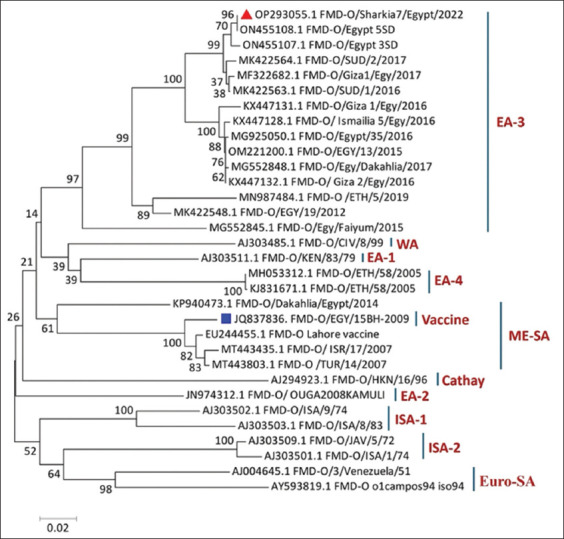
Phylogenetic analysis of the *VP1* coding sequences shows the relationship between different topotypes of FMDV serotype O. The tree was created using the Tamura-Nei model of nucleotide substitution with 1000 bootstrap replicates in MEGA11 software. The colored triangle represents the FMDV strain identified in this study. FMDV=Foot-and-mouth disease virus.

The phylogenetic analysis and *VP1*-coding sequences revealed that FMDV type O in this study was clustered in the East Africa-3 topotype (EA-3 topotype) (Figure 5). BLAST analysis revealed that the strain was 100% identical to the O/Egypt 5SD strain (Accession No. ON455108.1) and had 99.2% similarity with other strains circulating in Egypt (O/Egypt 3SD; ON455107.1 and O/Giza1/Egy/2017; MF322682.1). Furthermore, it exhibited a high genetic relationship with the Sudanese strains (O/SUD/1/2016; MK422563.1) and (O/SUD/2/2017; MK422564.1) with a 98.8% similarity. The genetic variance of the earlier Egyptian isolates in 2016 and 2017 (Ismailia 5/Egy 2016 and O/Egy/Dakahlia/2017) ranged from 5.1% to 5.3%. The Egyptian strain identified in 2012 (O/EGY/19/2012; MK422548.1) was closely linked to the Ethiopian strain (O/ETH/5/2019; MN987484), sharing a 95% genetic resemblance. Both variants displayed genetic similarity to current strain percentages of 92.5% and 90%, respectively. Our sequenced strain exhibited a 13.6% difference from the previously detected variant in Fayum in 2015 (O/Egy/Fayum/2015; MG552845.1) and differed from the vaccine strain (O/EGY/15BH-2009; JQ837836.1), which is attributed to the Middle East-South Asia topotype with an 18% variation in the nucleotide sequence.

**Figure 6:**
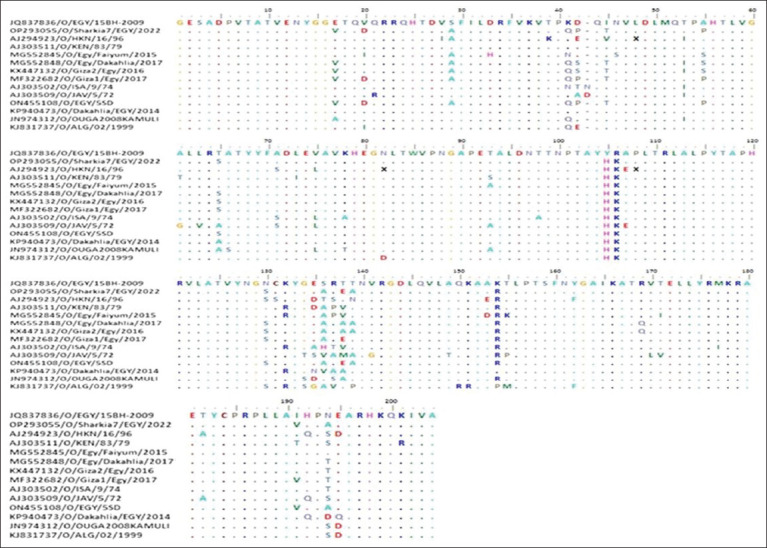
*VP1* amino acid variations between the FMDV serotype O strain identified in this study and the vaccination strain. The identity of the vaccine strain is indicated by dot-filled lines. FMDV=Foot-and-mouth disease virus.

The comparison of amino acid sequences showed several differences in the immunogenic G-H loop of our sequenced strain (O/Sharkia7/Egypt/2022; OP293055.1) compared with the FMD vaccine strain used in Egypt (O/EGY/15BH-2009; JQ837836.1), with an 8% genetic variation in the VP1 protein sequence (Figure 6). Our sequenced strain featured distinct amino acid substitutions: S136A, T138E, T139A, and K154R. The strain circulating in Egypt in 2022 (O/Egypt 5SD; ON455108.1) exhibited the same amino acid mutations as our sequenced strain.

## DISCUSSION

Aphthous fever ranks among the most contagious and economically impactful viral diseases affecting livestock health and productivity. In 2022, despite Egyptian efforts to control and eliminate the virus, multiple FMD outbreaks caused by serotype O (EA-3) and serotype A (Africa/G-IV) have occurred. New mutations of serotype A (EURO-SA topotype) and serotype O (Euro-SA topotype) have also been identified in Egypt [27, 28], despite our study not identifying FMDV with the same genetic makeup, which is likely due to the introduction of viruses through imported animals. Thus, it is important to continue surveillance to prevent viral outbreaks and monitor new FMDVs in Egypt [28].

The phylogenetic analysis of FMDV serotypes A and O showed that the vaccinal strains (A/EGY 1/2012 [KC440882.1] and O/EGY/15BH-2009 [JQ837836.1]) were grouped separately from the study strains and recently isolated reference strains. The divergence between our sequenced and vaccinal strains could explain why vaccinated animals are not fully protected against the recent variants of the virus. Similar conclusions were reported by Carrillo [29] and Abd-Ellatieff *et al*. [30], who emphasized the importance of the topotype classification system in selecting vaccines.

Our sequenced FMD-A strains closely resembled those from Sudan (A/SUD/6/2018; MK422591.1) and Ethiopia (A/ETH/19/2015; MN987497.1). Moreover, our sequenced FMD-O strain was closely associated with Sudanese strains (MK422563.1 and MK422564.1), indicating that both emerging viruses originated from the importation of live cattle from Sudan and Ethiopia. Our findings strongly supported the previous results by Al-Hosary *et al*. [31], who concluded that the isolated FMD-O strains showed significant similarity to previously reported Sudanese strains in 2008.

Amino acid variation analysis of the VP1 protein of FMDV serotype A viruses compared with the vaccine strain revealed that the VP1 protein exhibits 84.4% homology with the local vaccine strains (32 amino acid substitutions). Our findings strongly supported the previous results by Hassan *et al*. [32] regarding FMD-A (G/IV) isolates of the 2020 outbreaks, which reported high sequence variability of their studied strains when compared with the local vaccine strain. This may be attributed to the high variability of *VP1* among FMD capsid proteins. Notably, these mutations were predominantly observed in three distinct antigenic sites: the G-H loop (residues near the arginine-glycine-aspartic acid (RGD) motif), the C-terminal residues, and the BC loop (residues 43–45 and 48). Moreover, most of them were non-synonymous amino acid substitutions leading to changes in the exposed residue polarity (P40S, A41S, and T135G in the A/EGY/1/2012 vaccine and N137S in the A/IRN/1/2005 vaccine) or from negatively charged side chains to uncharged side chains (D138S in the A/EGY/1/2012 vaccine). Another comprehensive review of FMD-A protein structure [33] emphasized the significance of residue 142 (~138 in the studied FMD-A sequences) within the GH loop in the three-dimensional arrangement of the GH loop according to the type of amino acid at this site. This, in turn, significantly affects the interaction between *VP1* and other protein structures. Consequently, mutations at these crucial antigenic sites notably affect the vaccine’s effectiveness against the prevailing strains.

The amino acid sequence of the identified FMD serotype O exhibited 92.2% homology with the vaccine strain used in the study area (O/EGY/15BH-2009; JQ837836.1) with 16 amino acid substitutions. Most of these mutations were located in important antigenic positions in the G-H loop of the VP1 protein (Residue 130-160) near the RGD motif. These mutations may result in changes in the polarity of S137A and T140A or a transformation from an uncharged amino acid to a negatively charged amino acid (T139E). Other non-conservative mutations were found in the C-terminus (N195A) and BC loop (K42Q). These mutations in specific antigenic regions may cause structural changes and assist the viral evasion of the immune response triggered by the vaccine strain [34]. Similarly, Fernandez-Sainz *et al*. [35] asserted that alterations in amino acids in the immunogenic G-H loop might pose challenges for vaccination. Until 2022, Egypt had been using a domestically manufactured polyvalent vaccine, including O Pan-Asia II, A Iran 2005, A EGY 2012, SAT2 Ghb-12, and SAT2 Lib-12. However, the genetic and immunological differences between these vaccine strains and the newly circulating FMD strains suggest that the current vaccine strains may not adequately protect against field strains. Similar findings were previously reported by Abd El Rahman *et al*. [36] and Al-Ebshahy *et al*. [37], emphasizing the need to evaluate locally produced vaccines based on the identified serotypes and lineages to ensure effective cross-protection [38].

## CONCLUSION

This study provides critical insights into the genetic characterization and epidemiology of FMDV in Egyptian cattle during 2022. It confirmed the circulation of two predominant serotypes, A (Africa/G-IV) and O (EA-3), with a high prevalence of serotype A. Genetic analyses revealed significant divergence between the field strains and vaccine strains, particularly in the immunogenic G-H loop of the VP1 protein, which may explain reduced vaccine efficacy. The phylogenetic analysis highlighted close genetic relationships with strains from Sudan and Ethiopia, indicating potential transboundary transmission.

The study’s strengths include a comprehensive sampling of clinically infected cattle across two key provinces representing smallholder farming systems. It also employed detailed genetic analysis, including phylogenetic relationships and amino acid sequence variations, to identify critical mutations affecting vaccine efficacy. The integration of molecular diagnostics and sequence-based characterization provided robust and actionable data.

However, there are some limitations. The study was geographically limited to Sharkia and Dakahlia provinces, potentially excluding variations in other regions of Egypt. A relatively small number of sequenced samples may limit the representativeness of genetic diversity among circulating FMDV strains. The lack of direct experimental validation of vaccine efficacy against the identified field strains constrains the practical implications of the findings.

Future research should expand geographically to include more regions and livestock systems to capture a broader genetic diversity of FMDV. Studies should focus on developing and validating updated vaccines tailored to the circulating strains, with experimental trials to assess cross-protection. In addition, enhanced surveillance and molecular epidemiological studies are needed to monitor FMDV evolution, particularly in light of transboundary animal movements. Strengthened biosecurity measures and vaccination strategies should also be explored to mitigate FMD’s economic and health impacts on vulnerable smallholder farming systems. This work underscores the urgent need for targeted interventions and regular updates to control strategies to address the dynamic nature of FMDV in Egypt.

## DATA AVAILABILITY

The data generated during the study is available in the article. There are no other supplementary data.

## AUTHORS’ CONTRIBUTIONS

SGY and HME: Study conception and design and drafted, reviewed, and edited the manuscript. HAE, YAE, EM, and SA: Conceived and designed the study. SGY, HAE, and YAE: Performed field and laboratory work. EM and SA: Data analysis and interpretation. All authors have read and approved the final manuscript.
